# Supplementary material for: Improving outcomes for neonates with gastroschisis in low-income and middle-income countries: a systematic review protocol
Source: BMJ Paediatr Open. 2018 Dec 27;2(1):e000392. doi: 10.1136/bmjpo-2018-000392 (PMC6326322; doi:10.1136/bmjpo-2018-000392)
Supplement: Supplementary file 2 [file bmjpo-2018-000392supp002.pdf]

Supplementary File 2

Improving outcomes for neonates with gastroschisis in low- and middle-income countries: a systematic review protocol

Database(s): **Ovid MEDLINE(R) In-Process & Other Non-Indexed Citations and Ovid MEDLINE(R)** 1946 to Present

#	Searches	Results
1	exp Infant, Newborn/	564962
2	(neonate* or newborn*).mp.	723943
3	1 or 2	726361
4	anorectal malformations/	499
5	anus, imperforate/	2460
6	esophageal atresia/	3337
7	intestinal atresia/	1996
8	hernias, diaphragmatic, congenital/	4294
9	gastroschisis/	1128
10	duodenal obstruction/	3179
11	intestinal volvulus/	2298
12	Gastroschisis/	1128
13	Anorectal Malformations/	499
14	Anus, Imperforate/	2460
15	Duodenal Obstruction/	3179
16	Intestinal Atresia/	1996
17	Intestinal Volvulus/	2298
18	Hernias, Diaphragmatic, Congenital/	4294
19	Esophageal Atresia/	3337
20	Tracheoesophageal Fistula/	3467
21	("congenital anomal*" or "congenital abnormal*" or "birth defect*" or gastroschisis or exomphalos or omphaloc?ele or "abdominal wall defect" or "anorectal malformation" or "imperforate anus" or "duodenal atresia" or "colon* atresia" or "intestin* atresia" or malrotation or "congenital diaphragmatic hernia*" or "?esophageal atresia" or "tracheo-?esophageal fistula" or volvulus or "congenital malformation*" or "jejunal atresia" or "jejuno-ileal atresia" or "ileal atresia" or "Hirschsprung's disease" or aganglionosis or "apple peel syndrome*" or "anorectal stenos?s" or "anal atresia*").mp.	98434
22	or/4-21	104428
23	Developing Countries/	69831

24	exp Africa/	233166
25	exp Asia, Central/	6779
26	exp Transcaucasia/	3966
27	exp Central America/	14334
28	Afghanistan/	2857
29	Albania/	762
30	American Samoa/	159
31	Argentina/	13689
32	Bangladesh/	9034
33	Bhutan/	343
34	Bolivia/	2275
35	"Bosnia and Herzegovina"/	1847
36	Brazil/	76758
37	Bulgaria/	6069
38	Cambodia/	2835
39	China/	132950
40	Comoros/	260
41	Croatia/	6164
42	Cuba/	4750
43	"Democratic People's Republic of Korea"/	184
44	Dominica/	84
45	Ecuador/	3085
46	Fiji/	834
47	Grenada/	117
48	Guyana/	621
49	Haiti/	2834
50	India/	91150
51	Indonesia/	8786
52	Iran/	20716
53	Iraq/	4218
54	Jamaica/	3295
55	Jordan/	3484

56	Kosovo/	124
57	Lebanon/	3590
58	Laos/	1634
59	Madagascar/	3026
60	Malaysia/	12977
61	Mauritius/	510
62	Mexico/	33915
63	Micronesia/	1074
64	Moldova/	642
65	Mongolia/	1532
66	Montenegro/	159
67	Myanmar/	1892
68	Nepal/	6570
69	Pakistan/	14361
70	Papua New Guinea/	3214
71	Paraguay/	691
72	Peru/	7366
73	Philippines/	7578
74	"Republic of Belarus"/	1992
75	Romania/	9348
76	Russia/	36547
77	Saint Lucia/	64
78	"Saint Vincent and the Grenadines"/	46
79	Samoa/	295
80	Serbia/	2531
81	Sri Lanka/	5179
82	Suriname/	855
83	Syria/	1400
84	Thailand/	23569
85	Timor-Leste/	148
86	Turkey/	29917
87	Ukraine/	15218

88	Vanuatu/	321
89	Venezuela/	4626
90	Vietnam/	10611
91	Yemen/	1234
92	(afghanistan or albania or algeria or "american samoa" or angola or argentina or armenia or azerbaijan or bangladesh or belarus or belize or benin or bhutan or bolivia or bosnia or botswana or brazil or bulgaria or "burkina faso" or burundi or "cabo verde" or "cape verde" or cambodia or cameroon or "central africa* republic" or chad or china or columbia or comoros or "costa rica" or "cote d'ivoire" or croatia or cuba or "democratic people's republic of korea" or "democratic republic of the congo" or djibouti or dominica or "dominica republic" or drc or "east timor" or eritrea or ecuador or "el salvador" or egypt or ethiopia or fiji or gabon or gambia or gaza or republic georgia or ghana or grenada or guatemala or guinea or "guinea-bissau" or guyana or haiti or herzegovina or honduras or india or indonesia or iran or "ivory coast" or jamaica or jordan or kazakhstan or kenya or kiribati or kosovo or "kyrgyz republic" or "lao pdr" or laos).mp.	825783
93	(lebanon or lesotho or liberia or libya or macedonia or madagascar or malawi or malaysia or maldives or mali or "marshall islands" or mauritania or mauritius or mexico or micronesia or moldova or mongolia or montenegro or morocco or mozambique or myanmar or namibia or nauru or nepal or nicaragua or nigeria or "timor-leste" or iraq or niger or panama or pakistan or "papua new guinea" or paraguay or peru or philippines or romania or russia or "russian federation" or rwanda or ruanda or "saint lucia" or "saint vincent and the grenadines" or samoa or "sao tome and principe" or senegal or senegambia or serbia or "sierra leone" or "solomon islands" or somalia or somaliland or "south africa" or "sri lanka" or "st lucia" or "st vincent and the grenadines" or "sub-saharan africa" or sudan or suriname or syria or "syrian arab republic" or swaziland or tajikistan or tanzania or thailand or togo or tonga or tunisia or turkey or turkmenistan or tuvalu or uganda or ukraine or uzbekistan or vanuatu or venezuela or vietnam or "west bank" or yemen or yugoslavia or zaire or zambia or zimbabwe).mp.	531582
94	(kabul or Porto-Novo or Hogbonou or Adjace or Cotonou or Kutonu or Ouagadougou or Ouaga or Bujumbura or Usumbura or Phnom Penh or Bangui or Bangi or N'Djamena or Ndjamena or Fort Lamy or Moroni or Kinshasa or Asmara or Asmera or Addis Ababa or Addis Abeba or Banjul or Bathurst or Conakry or Bissau or Port-au-Prince or Pyongyang or Monrovia or Antananarivo or Tananarive or Tana or Lilongwe or Bamako or Maputo or Lourenco Marques or Kathmandu or Niamey or Kigali or Freetown or Free-town or Mogadishu or Xamar or Hamar or Muqdisho or Maqadishu or Juba or Dodoma or Dar es Salaam or Lome or Kampala or Harare or Salisbury or Yerevan or Dhaka or Dacca or Thimphu or Thimbu or Sucre or Charcas or La Plata or Chuquisaca or La Paz or Praia or Yaounde or Jaunde or Brazzaville or Yamoussoukro or Cairo or Accra).mp.	24386
95	(Tegucigalpa or Tegus or New Delhi or Jakarta or Nairobi or South Tarawa or Tarawa Teinainano or Pristina or Prishtina or Bishkek or Pishpek or Frunze or Vientiane or Maseru or Nouakchott or Palikir or Chisinau or Kishinev or Rabat or Nay Pyi Taw or Naypyidaw or Nepranytau or Naypyitaw or Kyetpyay or Pyinmana or Kyatpyay or Pyinmana or Yangon or Rangoon or Managua or Abuja or Lagos or Islamabad or Port Moresby or Moresby or Pom Town or Manila or Apia or Dakar or Honiara or Jayawardenepura or Jayewardenepura or Khartoum or Mbabane or Embabane or Lobamba or Damascus or Dushanbe or Dyushambe or Stalinabad or Dili or Kyiv or Kiev or Tashkent or Toshkent or Port Vila or Hanoi or Ha Noi or Sana'a or Sanaa or Sana or Lusaka or Ulaanbaatar or Ulan-Bator or Luanda or Tbilisi or Amman).mp.	21747
96	(("under developed" or underdeveloped or low income or middle income or developing or less developed or "third world" or poor or LMI or LLMI or LAMI) adj1 (countr* or nation* or economy or economies)).mp.	128848
97	(("resource limited" or "low resource") adj1 (countr* or setting*)).mp.	7852
98	("less resourced communit*" or LMIC or LMICs).mp.	2416
99	or/23-98	1391627
100	3 and 22 and 99	2565
101	exp animals/ not humans/	4448581
102	100 not 101	2551
