# Supplementary material for: Improving outcomes for neonates with gastroschisis in low-income and middle-income countries: a systematic review protocol
Source: BMJ Paediatr Open. 2018 Dec 27;2(1):e000392. doi: 10.1136/bmjpo-2018-000392 (PMC6326322; doi:10.1136/bmjpo-2018-000392)
Supplement: Author's manuscript [file bmjpo-2018-000392.draft_revisions.pdf]

BMJ Paediatrics Open

BMJ Paediatrics Open is committed to open peer review. As part of this commitment we make the peer review history of every article we publish publicly available.

When an article is published we post the peer reviewers' comments and the authors' responses online. We also post the versions of the paper that were used during peer review. These are the versions that the peer review comments apply to.

The versions of the paper that follow are the versions that were submitted during the peer review process. They are not the versions of record or the final published versions. They should not be cited or distributed as the published version of this manuscript.

BMJ Paediatrics Open is an open access journal and the full, final, typeset and author-corrected version of record of the manuscript is available on our site with no access controls, subscription charges or pay-per-view fees (http://bmjpaedsopen.bmj.com).

If you have any questions on BMJ Paediatrics Open's open peer review process please email info.bmjpo@bmj.com

BMJ Paediatrics Open

Confidential: For Review Only

Improving outcomes for neonates with gastroschisis in low- and middle-income countries: a systematic review protocol

Journal:	*BMJ Paediatrics Open*
Manuscript ID	bmjpo-2018-000392
Article Type:	Protocol
Date Submitted by the Author:	10-Oct-2018
Complete List of Authors:	Wright, Naomi; King's Centre for Global Health and Health Partnerships<br>Langer, Monica; Northwestern University<br>Norman, Irena; GKT School of Medical Education<br>Akhbari, Melika; GKT School of Medical Education<br>Wafford, Q. Eileen; Northwestern University<br>Ade-Ajayi, Niyi; King's College Hospital, Department of Paediatric Surgery<br>Davies, Justine; King's Centre for Global Health and Health Partnerships<br>Poenaru, Dan ; McGill University<br>Sevdalis, Nick; King's College London<br>Leather, Andy; King's Centre for Global Health and Health Partnerships
Keywords:	Paediatric Surgery, Congenital Abnorm, Clinical Procedures, Intensive Care, Nursing Care

SCHOLARONE™
Manuscripts

Improving outcomes for neonates with gastroschisis in low- and middle-income countries: a systematic review protocol

Naomi J Wright[1], Monica Langer[2], Irena CF Norman[3], Melika Akhbari[3], Q. Eileen Wafford[2], Niyi Ade-Ajayi[4], Justine Davies[1], Dan Poenaru[5], Nick Sevdalis[6], Andy Leather[1]

[1] King's Centre for Global Health and Health Partnerships, School of Population Health and Environmental Sciences, King's College London, UK
[2] Northwestern University, Chicago, USA
[3] GKT School of Medical Education, King's College London, UK
[4] Paediatric Surgery Department, King's College Hospital, London, UK
[5] McGill University, Montreal, Canada
[6] Centre for Implementation Science, King's College London, UK

Author email addresses:
naomiwright@doctors.org.uk, langerm7@hotmail.com, irena.norman@kcl.ac.uk, melika.akhbari@kcl.ac.uk, q-wafford@northwestern.edu, niyi.ade-ajayi@nhs.net, Justine.davies@kcl.ac.uk, nick.sevdalis@kcl.ac.uk, andy.leather@kcl.ac.uk

Corresponding author:
Miss Naomi Wright MBChB (Hons) BSc (Hons) MRCS DCH MSc
Paediatric Surgery Registrar and Wellcome Trust Clinical PhD Fellow in Global Health
King's Centre for Global Health and Health Partnerships, Suite 2.13, Weston Education Centre, Cutcombe Road, London, SE5 9RJ.

PROSPERO Registration: CRD42018095349
Registered 3rd July 2018

Protocol prepared using PRISMA-P Guidelines

Confidential: For Review Only

ABSTRACT

Introduction: There is a significant disparity in outcomes for neonates with gastroschisis in high-income countries (HICs) compared to low- and middle-incomes countries (LMICs). Many LMICs report mortality rates between 75-100% compared to less than 4% in HICs.

Aim: To undertake a systematic review identifying postnatal interventions associated with improved outcomes for gastroschisis in LMICs.

Methods and analysis: Three search strings will be combined: 1) neonates; 2) gastroschisis and other gastrointestinal congenital anomalies requiring similar surgical care; 3) LMICs. Databases to be searched include: MEDLINE, EMBASE, Scopus, Wed of Science, ProQuest Dissertations and thesis global and the Cochrane Library. Grey literature will be identified through Open-Grey, ClinicalTrials.gov, WHO International Clinical Trials Registry and ISRCTN registry (Springer Nature). Additional studies will be sought from reference lists of included studies. Study screening, selection, data extraction and assessment of methodological quality will be undertaken by two reviewers independently and team consensus sought on discrepancies.

The primary outcome will be mortality. Secondary outcomes include: complications, requirement for ventilation, parenteral nutrition duration and length of hospital stay. Tertiary outcomes include service delivery and implementation outcomes. The methodology of the studies will be appraised. Descriptive statistics and outcomes will be summarised and discussed.

Ethics & dissemination: Ethical approval is not required since no new data is being collected. Dissemination will be via open access publication in a peer-reviewed medical journal and distribution amongst global health, global surgery and children's surgical collaborations and international conferences.

Conclusion: This study will systematically review literature focussed on postnatal interventions to improve outcomes from gastroschisis in LMICs. Results can be used to help enhance clinical practice in low-resource settings and inform interventional studies aimed at improving outcomes for gastroschisis and other similar congenital anomalies involving the gastrointestinal tract.

Confidential: For Review Only

What is already known on this topic?

- Congenital anomalies are the 5th leading cause of death in children under 5-years of age globally.
- Gastroschisis is one of the commonest congenital anomalies with an increasing global incidence.
- Mortality due to gastroschisis is above 75% in many low- and middle-income countries (LMICs) compared to 4% in high-income countries.

What this study hopes to add:

- To systematically identify studies that evaluate postnatal interventions aimed at improving outcomes for neonates with gastroschisis in LMICs.
- To identify interventions used in the management of other congenital anomalies involving the gastrointestinal tract that are transferrable to the treatment of gastroschisis in LMICs.

Confidential: For Review Only

INTRODUCTION

Congenital anomalies are estimated to be the fifth leading cause of death in under 5-year olds globally (1). Gastroschisis (a condition where the intestines protrude through a hole in the abdominal wall at birth) is one of the commonest congenital anomalies and has been increasing in incidence (2-6). Since the 1960s mortality from gastroschisis has fallen in high-income countries (HICs) to less than 5% today (7). Mortality has fallen to a lesser extent in middle-income countries. Recent literature reports mortality rates of: 80%, Iran; 36%, Turkey; and 6-8%, Thailand (8-11). In low-income countries mortality remains high, with many sub-Saharan African countries reporting mortality rates of 75 - 100% (12-15).

Management of gastroschisis varies widely. The most common interventions in HICs are primary closure in the operating room or use of a preformed silo with gradual intestinal reduction and delayed closure, often at the cotside without general anaesthetic (16). Systematic reviews report comparable outcomes for both methods in HICs, but preformed silos are associated with lower ventilation requirements (17, 18). Preformed silo use has additional benefits for low- and middle-income countries (LMICs): it is low-technology; avoids neonatal anaesthesia and surgery; can be applied at the cotside by any trained healthcare personnel and reduces intensive care requirements due to lower intra-abdominal pressures (17). This is advantageous in low-resource settings where there is variable availability of paediatric surgeons, deficient intensive care facilities and safety of neonatal anaesthesia and surgery is limited by lack of trained staff and resources (19,20).

However, preformed silos are expensive and have been largely unavailable in LMICs (12). Alternative strategies have been devised with varying success (12). Examples include use of

an Alexis Wound Retractor or improvised surgical silo, primary reduction at the cotside (Bianchi technique) and umbilical turban and flap closure (21-28). Antenatal diagnosis, delivery in a tertiary paediatric surgery centre, pre-hospital management, neonatal resuscitation and nutrition are also important components of care that may impact on outcomes (15).

The focus of this systematic review is to identify postnatal pre-hospital and in-hospital interventions aimed at improving outcomes for neonates with gastroschisis in LMICs. In this review, an 'intervention' is defined as any action taken to improve a patient's medical condition. This includes specific interventions for gastroschisis and generic interventions used for a wider range of congenital anomalies involving the gastrointestinal tract, which may also be beneficial for patients with gastroschisis.

METHODS AND ANALYSIS

Preferred reporting items for systematic review and meta-analysis (PRISMA-P) 2015 guidelines have been followed in this protocol (29). If amendments to the protocol occur, they will be reported in the publication of the results.

Aim

To identify postnatal pre-hospital and in-hospital interventions associated with improved outcomes for neonates with gastroschisis in LMICs.

Objectives

1) To identify studies that evaluate postnatal interventions to improve outcomes for neonates with gastroschisis in LMICs.

2) To identify generic surgical care interventions used in LMICs to manage neonates with a wider range of structural congenital anomalies involving the gastrointestinal tract, which may be transferrable to the care of neonates with gastroschisis.

3) To critically appraise the methodological quality of the evidence.

4) To provide an evidence-based summary of the condition-specific and generic neonatal surgical care interventions associated with improved outcomes for gastroschisis in LMICs to inform clinical practice and future studies.

Search strategy

Three search strings will be combined to identify studies that evaluate postnatal interventions aimed at improving outcomes in neonates with gastroschisis or other structural congenital anomalies involving the gastrointestinal tract in LMICs. The search

Confidential: For Review Only

strings include: 1) the population - neonates, 2) the conditions - gastroschisis or other structural congenital anomalies involving the gastrointestinal tract, 3) the context - LMICs.

Neonates are defined as infants within the first 28-days of life. Inclusion criteria for structural congenital anomalies involving the gastrointestinal tract are outlined in search string 2 below. These were derived from consensus amongst the authors according to what conditions may utilise similar neonatal surgical care as gastroschisis and thus have relevant transferrable interventions. The third string incorporates all terminology utilised to describe LMICs and will include searching for all studies published from countries listed as low- or middle-income by the World Bank in 2018 (30). Individual countries and major cities will be included.

The search terms to be utilised are listed in Table 1. Within each search string, terms will be combined using 'OR'. The three strings will then be combined using 'AND'. Truncation and wildcards will be utilised to incorporate all term variations. Medical Subject Headings (MeSH) terms will be utilised. Phrase and adjacency searching will be utilised to focus the search on the required terms. Applied proximity searching using the adjacency operator (ADJ1) will be used to capture phrasing variations. If additional key terms are identified during the search, they will be added to the appropriate string to ensure it is maximally inclusive. An example of this search strategy applied to MEDLINE can be found in Supplementary Document 1. This strategy will be adapted to the other databases.

Confidential: For Review Only

Table 1: Three search strings to be utilised to identify studies to be included in the systematic review

**Search String 1**	**Search String 2**	**Search String 3**
newborn<br>neonate	congenital anomalies, congenital abnormalities, congenital malformation, birth defects, gastroschisis, exomphalos, omphalocele, abdominal wall defect, intestinal atresia, apple peel syndrome, duodenal atresia, duodenal obstruction, duodenal web, jejunal atresia, jejuno-ileal atresia, ileal atresia, colonic atresia, anorectal malformation, anorectal stenosis, imperforate anus, anal atresia, malrotation, volvulus, congenital diaphragmatic hernia, oesophageal atresia, tracheo-oesophageal fistula, Hirschsprung's Disease, Aganglionosis	low income countries,<br>middle income countries,<br>LMICs, LAMI, LMI<br>low resource settings, resource limited setting, less resourced communities, developing countries, underdeveloped countries, third world countries, developing nations, low income nation,<br>sub-Saharan Africa,<br>Afghanistan, Albania, Algeria, American Samoa, Angola, Argentina, Armenia, Azerbaijan, Bangladesh, Belarus, Belize, Benin, Bhutan, Bolivia, Bosnia and Herzegovina, Botswana, Brazil, Bulgaria, Burkina Faso, Burundi, Cabo Verde, Cambodia, Cameroon, Central African Republic, Chad, China, Columbia, Comoros, Democratic Republic of the Congo, DRC, Republic of the Congo, Costa Rica, Cote d'Ivoire, Ivory Coast, Croatia, Cuba, Djibouti, Dominica, Ecuador, Egypt, El Salvador, Equatorial Guinea, Eritrea, Ethiopia, Fiji, Gabon, Gambia, Georgia, Ghana, Grenada, Guatemala, Guinea, Guinea-Bissau, Guyana, Haiti, Honduras, India, Indonesia, Islamic Republic of Iran, Iraq, Jamaica, Jordan, Kazakhstan, Kenya, Kiribati, Democratic People's Republic of Korea, Kosovo, Kyrgyz Republic, Lao PDR, Laos, Lebanon, Lesotho, Liberia, Libya, Macedonia Republic, Madagascar, Malawi, Malaysia, Maldives, Mali, Marshall Islands, Mauritania, Mauritius, Mexico, Micronesia, Moldova, Mongolia, Montenegro, Morocco, Mozambique, Myanmar, Namibia, Nauru, Nepal, Nicaragua, Niger, Nigeria, Pakistan, Panama, Papua New Guinea, Paraguay, Peru, Philippines, Romania, Russian Federation, Rwanda, Samoa, Sao Tome and Principe, Senegal, Serbia, Sierra Leone, Solomon Islands, Somalia, Somaliland, South Africa, Sri Lanka, St. Lucia, Saint Lucia, St. Vincent and Grenadines, Saint Vincent and the Grenadines, Sudan, Suriname, Swaziland, Syrian Arab Republic, Syria, Tajikistan, Tanzania, Thailand, Timor-Leste, East Timor, Togo, Tonga, Tunisia, Turkey, Turkmenistan, Tuvalu, Uganda, Ukraine, Uzbekistan, Vanuatu, Venezuela, Vietnam, West Bank and Gaza, Republic of Yemen, Zambia, Zimbabwe.<br>Kabul, Porto-Novo, Hogbonou, Adjace, Cotonou, Kutonu, Ouagadougou, Ouaga, Bujumbura, Usumbura, Phnom Penh, Bangui, Bangi, N'Djamena, Ndjamena, Fort Lamy, Moroni, Kinshasa, Asmara, Asmera, Addis Ababa, Addis Abeba, Banjul, Bathurst, Conakry, Bissau, Port-au-Prince, Pyongyang, Monrovia, Antananarivo, Tananarive, Tana, Lilongwe, Bamako, Maputo, Lourenco Marques, Kathmandu, Niamey, Kigali, Freetown, Free-town, Mogadishu, Xamar, Hamar, Muqdisho, Maqadishu, Juba, Dodoma, Dar es Salaam, Lome, Kampala, Harare, Salisbury, Yerevan, Dhaka, Dacca, Thimphu, Thimbu, Sucre, Charcas, La Plata, Chuquisaca, La Paz, Praia, Yaounde, Jaunde, Brazzaville, Yamoussoukro, Cairo, Accra, Tegucigalpa, Tegus, New Delhi, Jakarta, Nairobi, South Tarawa, Tarawa Teinainano, Pristina, Prishtina, Bishkek, Pishpek, Frunze, Vientiane, Maseru, Nouakchott, Palikir, Chisinau, Kishinev, Rabat, Nay Pyi Taw, Naypyidaw, Nepranytau, Naypyitaw, Kyetpyay, Pyinmana, Kyatpyay, Pyinmana, Yangon, Rangoon, Managua, Abuja, Lagos, Islamabad, Port Moresby, Moresby, Pom Town, Manila, Apia, Dakar, Honiara, Jayawardenepura, Jayewardenepura, Khartoum, Mbabane, Embabane, Lobamba, Damascus, Dushanbe, Dyushambe, Stalinabad, Dili, Kyiv, Kiev, Tashkent, Toshkent, Port Vila, Hanoi, Ha Noi, Sana'a, Sanaa, Sana, Lusaka, Ulaanbaatar, Ulan-Bator, Luanda, Tbilisi, Amman

Confidential: For Review Only

Published literature search

The following databases will be searched using the search strategy above: MEDLINE (Ovid), EMBASE (Elsevier), Scopus (Elsevier), Web of Science (Clarivate Analytics), and the Cochrane Library. Language, year of publication or type of study will not be restricted. Only human studies will be included. The comprehensive field tag MP (multi-purpose) will be employed to search the following fields: Abstract, Protocol Supplementary Concept Word, Synonyms, Keyword Heading Word, Rare Disease Supplementary Concept Word, Title, Name of

Substance Word, Subject Heading Word, Unique Identifier and Original Title. Literature reviews and reference lists of included studies will be searched for further studies suitable for inclusion. All study types will be included, both published and unpublished.

Identification of grey literature

Grey literature will be identified through the following search engines: Open-Grey, ClinicalTrials.gov, WHO International Clinical Trials Registry and ISRCTN registry (Springer Nature). The following publication types will be included: dissertations/ theses, books/ book chapters, conference abstracts, editorials/ letters/ comments, newspapers/ trade journals, literature reviews, and research in progress. The authors of published conference proceedings will be contacted for a full report of data and findings where available. Experts in the field will be contacted to identify any ongoing research on this subject, which has yet to be published.

Inclusion/ exclusion criteria

Conditions

Conditions to be included are listed in column 2 of Table 1 (search string 2). Neonates with these structural congenital anomalies involving the gastrointestinal tract commonly present with a life-threatening emergency requiring a similar package of care within the neonatal period.

Structural congenital anomalies involving the gastrointestinal tract to be excluded from the search criteria include: biliary atresia, choledochal cyst and all other conditions not listed under search string 2. These conditions often present outside of the neonatal period.

Setting and participants

Studies containing preterm and term neonates presenting within the first 28-days of life with gastroschisis or one of the structural congenital anomalies listed in search string 2 will be included. Studies including just patients who have previously received care and re-presented with a complication or need for further intervention will be excluded. Only studies which have been undertaken in LMICs will be included.

Interventions

All pre-hospital and in-hospital postnatal interventions for the care of neonates with gastroschisis in LMICs will be included. Generic interventions related to the care of neonates with a structural congenital anomaly involving the gastrointestinal tract will be included. Antenatal interventions will be excluded because they are currently being evaluated in a separate systematic review.

Types of interventions will be categorised into specific interventions for neonates with gastroschisis and generic neonatal surgical care interventions for structural congenital

https://mc.manuscriptcentral.com/bmjpo
Confidential: For Review Only

anomalies involving the gastrointestinal tract. Generic interventions will be sub-categorised into: pre-hospital care and transportation, place of delivery, neonatal resuscitation and care, staffing, access to parenteral nutrition and other. Operative interventions related specifically and solely to a condition other than gastroschisis will be excluded. For example, operative techniques for oesophageal atresia or anorectal malformation.

Outcome measures

The primary outcome of the review will be mortality. This will include: all-cause in-hospital mortality, mortality within the neonatal period (within 28-days of life) and 30-day post-intervention mortality. Secondary outcomes will include: complications (post-primary intervention*), requirement for ventilation (yes/ no, number of days), duration of parenteral nutrition (days) and length of hospital stay (days). Complications will be determined using the Clavien-Dindo Classification, Table 2 (31).

*Primary intervention is defined as the first intervention the neonate received for bowel coverage, including application of a preformed silo at the cot-side.

Table 2: Clavien-Dindo Classification of Complications (31)

Grade	Definition
I	Any deviation from the normal post-operative course without the need for pharmacological treatment or surgical, endoscopic, and radiological interventions. Allowed therapeutic regimes are as follows: drugs as anti-emetics, anti-pyretics, analgesics, diuretics, electrolytes, parenteral nutrition, and physiotherapy. This grade also includes wound infections opened at the bedside.
II	Requiring pharmacological treatment with drugs other than such allowed for grade I complications. Blood transfusions are also included.
III	Requiring surgical, endoscopic, or radiological intervention.
IIIa	Intervention not under general anaesthesia.
IIIb	Intervention under general anaesthesia.
IV	Life-threatening complication requiring ICU management.
IVa	Single organ dysfunction (including dialysis).
IVb	Multi-organ dysfunction.
V	Death of a patient.

Tertiary outcomes include service delivery and implementation outcomes (Table 3). Implementation strategies will also be analysed. An implementation strategy is defined as the method(s) or technique(s) used to enhance the adoption, implementation, and sustainability of a clinical program or practice (32).

Confidential: For Review Only

Table 3: Definition of Implementation Outcomes (33)

Implementation outcome	Definition
Acceptability	Perception amongst stakeholders that the new intervention is agreeable.
Adoption	Intention to apply new intervention
Appropriateness	Perceived relevance of the intervention for the setting and problem
Feasibility	Extent to which an intervention can be applied
Fidelity	The proportion of management protocol components completed as intended.
Coverage	The proportion of eligible patients who actually receive the intervention.
Cost	Costs of the intervention, including the delivery strategy.
Sustainability	Extent to which a new intervention becomes routinely available/ is maintained post-introduction.

Study screening

References identified through the electronic search engines will be entered into Covidence and duplicates removed (34). Two reviewers will independently screen the abstracts of all references. All potentially relevant articles will have the full text reviewed in duplicate against the eligibility criteria. This will include the full text of articles in languages other than English, which will be translated. Any discrepancies or queries will be resolved by consensus with the wider authorship group. All reviewers are trained in systematic review methods. The search results will be represented using a PRISMA flow chart (35).

Data collection

Data will be extracted by two reviewers independently and entered into a pre-determined data collection form. Data will be collected on the study type, country, year of publication, journal of publication, authors' names, number of patients, patient demographics (including proportion with simple and complex gastroschisis*, gestational age, weight, time from birth to presentation at the study hospital and American Association of Anesthesiologists Score at the time of primary intervention), pre-hospital and in-hospital intervention(s), implementation strategy where relevant, primary and secondary clinical outcomes as detailed above, service delivery outcomes and implementation outcomes if available.

*Patients with bowel necrosis, perforation, atresia or closing/ closed gastroschisis.

Data synthesis

Descriptive statistics will be used to present the interventions and outcomes in results tables. Interventions will be categorised into gastroschisis-specific and generic neonatal surgical care, with the latter being further sub-categorised as detailed above. Because a wide range of interventions and outcomes will be evaluated, it is unlikely that a meta-

Confidential: For Review Only

analysis will be feasible. However, if there is appropriate data, a meta-analysis will be undertaken.

Methodological quality appraisal and bias assessment

The methodological quality of the studies will be assessed and the findings summarised in a table to aid interpretation. Cochrane Risk of Bias for Non-Randomised Studies of Interventions (ROBINS-I) and the revised tool for Risk of Bias in randomised trials (RoB 2.0) will be used to assess quantitative studies (36, 37). This will be undertaken by two reviewers independently and team consensus sought for discrepancies.

DISCUSSION

To our knowledge, this will be the first systematic review focussed on postnatal interventions to improve outcomes from gastroschisis in LMICs. Such a review is vital to address outcome disparity, with many neonates with gastroschisis dying in LMICs and the majority surviving in HICs (12, 15). It is hoped lessons learned in centres with better outcomes within LMICs can be evaluated and shared amongst the global community to improve outcomes and inform future interventional studies. A wider range of congenital anomalies involving the gastro-intestinal tract will be incorporated into the study to help identify generic neonatal surgical care interventions that have the potential to improve outcomes for neonates with gastroschisis. This information may also help to inform clinical practice for a wider range of structural congenital anomalies involving the gastrointestinal tract. The systematic review may also highlight areas for improvement in HICs, such as cost reduction.

Strengths and limitations

This study is unique in its focus and comprehensive search strategy incorporating both original articles, grey literature, published and unpublished work. Incorporating a wider range of structural congenital anomalies within the search strategy will help identify neonatal surgical care interventions utilised in LMICs that could be beneficial for patients with gastroschisis. Identified articles in languages other than English will be translated so they can be included within the review.

Although the search strategy has been designed to be optimally inclusive, it is possible that articles could be missed. The initial search will be undertaken in English and some articles without English translation of the title or abstract could be missed. This systematic review will only include studies undertaken within LMICs; it may be possible that low-technology interventions utilised within HICs could also benefit gastroschisis care in low-resource settings.

Confidential: For Review Only

Ethics and dissemination

This systematic review will analyse previously published historical data, thus does not require ethical approval.

The results will be submitted for open access publication in a peer-reviewed medical journal. The publication will be disseminated amongst the PaedSurg Africa Research Collaboration and Global PaedSurg Research Collaboration consisting of hundreds of children's surgical care providers across the globe (38). It will also be shared amongst members of the Global Initiative for Children's Surgery (GICS), which includes all members of the multi-disciplinary team caring for neonates with gastroschisis and international organisations, policy makers and representatives from the World Health Organisation (39, 40). Results will be disseminated using social media. Findings will be presented internationally with a focus on global health, global surgery, paediatric and paediatric surgical conferences in LMICs.

The results will help to inform the development of an interventional care bundle to be evaluated in a Wellcome Trust funded multi-centre interventional study aimed at improving survival in neonates with gastroschisis in LMICs. This will be undertaken in seven tertiary paediatric surgery centres across sub-Saharan Africa between 2018 - 2020.

Acknowledgements

We thank the library services at King's College London, UK; Northwestern University, Chicago, USA; and Elena Guadagno, MLIS, and Alex Amar, MLIS, at McGill University Health Centre (MUHC), Montreal, Canada for their help with the literature search.

Confidential: For Review Only

REFERENCES

1. Global Burden of Disease (GBD) Child Mortality Collaborators. Global, regional, national, and selected subnational levels of stillbirths, neonatal, infant, and under-5 mortality, 1980-2015: a systematic analysis for the Global Burden of Disease Study 2015. *Lancet* 2016;388(10053):1725-74.
2. Agugua NE, Nwako FA. Gastroschisis a fifteen-year experience. *West Afr J Med* 1990;9(2):147-50.
3. Alvarez SM, Burd RS. Increasing prevalence of gastroschisis repairs in the United States: 1996-2003. *J Pediatr Surg* 2007;42(6):943-6.
4. Arnold M. Is the incidence of gastroschisis rising in South Africa in accordance with international trends? A retrospective analysis at Pretoria Academic and Kalafong Hospitals, 1981-2001. *S Afr J Surg* 2004;42(3):86-8.
5. Benjamin BG, Ethen MK, Van Hook CL, et al. Gastroschisis prevalence in Texas 1999-2003. *Birth Defects Res A Clin Mol Teratol* 2010;88(3):178-85.
6. Tan KB, Tan KH, Chew SK et al. Gastroschisis and omphalocele in Singapore: a ten-year series from 1993 to 2002. *Singapore Med J* 2008;49(1):31-6.
7. Bradnock TJ, Marven S, Owen A et al. Gastroschisis: one year outcomes from national cohort study. *BMJ* 2011;343:d6749.
8. Askarpour S, Ostadian N, Javaherizadeh H et al. Omphalocele, gastroschisis: epidemiology, survival, and mortality in Imam Khomeini hospital, Ahvaz-Iran. *Pol Przegl Chir* 2012;84(2):82-5.
9. Erdogan D, Azili MN, Cavusoglu YH et al. 11-year experience with gastroschisis: factors affecting mortality and morbidity. *Iran J Pediatr* 2012;22(3):339-43.
10. Niramis R, Suttiwongsing A, Buranakitjaroen V et al. Clinical outcome of patients with gastroschisis: what are the differences from the past? *J Med Assoc Thai* 2011;94 Suppl 3:S49-56.
11. Sangkhathat S, Patrapinyokul S, Chiengkriwate P et al. Infectious complications in infants with gastroschisis: an 11-year review from a referral hospital in southern Thailand. *J Pediatr Surg* 2008;43(3):473-8.
12. Wright NJ, Zani A, Ade-Ajayi N. Epidemiology, management and outcome of gastroschisis in Sub-Saharan Africa: Results of an international survey. *Afr J Paediatr Surg* 2015;12(1):1-6.
13. Wesonga AS, Fitzgerald TN, Kabuye R et al. Gastroschisis in Uganda: Opportunities for improved survival. *J Pediatr Surg* 2016;51(11):1772-7.
14. Apfeld JC, Wren SM, Macheka N et al. Infant, maternal, and geographic factors influencing gastroschisis related mortality in Zimbabwe. *Surgery* 2015;158(6):1475-80.
15. Ford K, Poenaru D, Moulot O et al. Gastroschisis: Bellwether for neonatal surgery capacity in low resource settings? *J Pediatr Surg* 2016;51(8):1262-7.
16. Zani A, Ruttenstock E, Davenport M et al. Is there unity in Europe? First survey of EUPSA delegates on the management of gastroschisis. *Eur J Pediatr Surg* 2013;23(1):19-24.
17. Ross AR, Eaton S, Zani A et al. The role of preformed silos in the management of infants with gastroschisis: a systematic review and meta-analysis. *Pediatr Surg Int* 2015;31(5):473-83.
18. Pastor AC, Phillips JD, Fenton SJ et al. Routine use of a SILASTIC spring-loaded silo for infants with gastroschisis: a multicentre randomized controlled trial. *J Pediatr Surg* 2008;43:1807-12.

Confidential: For Review Only

19. Bosenberg AT. Neonatal anesthesia with limited resources. *Paediatr Anaesth* 2014;24(1):98-105.
20. Krishnaswami S, Nwomeh BC, Ameh AE. The pediatric surgery workforce in low- and middle-income countries: problems and priorities. *Semin Pediatr Surg* 2016;25(1):32-42.
21. Emami CN, Youssef F, Baird RJ et al. A risk-stratified comparison of fascial versus flap closure techniques on the early outcomes of infants with gastroschisis. *J Pediatr Surg* 2015;50(1):102-6.
22. Duncan ND, Brown B, Dundas SE et al. "Minimal intervention management" for gastroschisis: a preliminary report. *West Indian Med J* 2005;54(2):152-4.
23. Ferreira C, G., Lacreuse I, Geslin D et al. Staged gastroschisis closure using Alexis wound retractor: first experiences. *Pediatr Surg Int* 2014;30:305-11.
24. Sandler A, Lawrence J, Meehan J. A "plastic" sutureless abdominal wall closure in gastroschisis. *J Pediatr Surg* 2004;39(5):738-41.
25. Kanamori Y. Operative general surgery in neonates and infants. Gastroschisis and Omphalocele. Taguchi T, editor: Springer Japan; 2016.
26. Kusafuka J, Yamataka A, Okazaki T et al. Gastroschisis reduction using 'Applied Alexis", a wound protector and retractor. *Pediatr Surg Int* 2005;21:925-7.
27. Ogasawara Y, Okazaki T, Kato Y et al. Spontaneous sutureless closire of the abdominal wall defect in gastroschisis using a commercial wound retractor system. *Pediatr Surg Int* 2009;25:973-6.
28. Bisaliev BN, Tsap NA. [Experience of Treatment of Newborn Children with Gastroschisis]. *Vestn Khir Im I I Grek* 2015;174(6):46-51.
29. Shamseer L, Moher D, Clarke M et al. Preferred reporting items for systematic review and meta-analysis protocols (PRISMA-P) 2015: elaboration and explanation. *BMJ* 2015;350:g7647.
30. World Bank. World Bank Country and Lending Groups. 2018. https://datahelpdesk.worldbank.org/knowledgebase/articles/906519-world-bank-country-and-lending-groups (Accessed 16th April 2018).
31. Clavien PA, Barkun J, de Oliveira ML et al. The Clavien-Dindo classification of surgical complications: five-year experience. *Ann Surg* 2009;250(2):187-96.
32. Proctor EK, Powell BJ, McMillen JC. Implementation strategies: recommendations for specifying and reporting. *Implement Sci* 2013;8:139.
33. Proctor E, Silmere H, Raghavan R et al. O
utcomes for Implementation Research: Conceptual Distinctions, Measurement Challenges, and Research Agenda. *Adm Policy Ment Health* 2011;38:65-76.
34. Cochrane. Covidence. www.covidence.org (Accessed 16th April 2018).
35. Moher D, Liberati A, Tetzlaff J et al. Preferred reporting items for systematic reviews and meta-analyses: the PRISMA statement. *J Clin Epidemiol* 2009;62(10):1006-12.
36. Higgins J, Sterne J, Savovic J et al. A revised tool for assessing risk of bias in randomized trials. *Cochrane Database Syst Rev* 2016;10(Supplement 1).
37. Sterne JA, Hernan MA, Reeves BC et al. ROBINS-I: a tool for assessing risk of bias in non-randomised studies of interventions. *BMJ* 2016;355:i4919.
38. Global PaedSurg Research Collaboration. www.globalpaedsurg.com (Accessed 16th April 2018).
39. Goodman LF, St-Louis E, Yousef Y et al. The Global Initiative for Children's Surgery: Optimal Resources for Improving Care. *Eur J Pediatr Surg* 2017.

Confidential: For Review Only

40. Global Initiative for Children's Surgery (GICS). www.globalchildrenssurgery.org (Accessed 16th April 2018).

Confidential: For Review Only

Contributions:

NW and ML conceived the idea for the study and designed the protocol. NW drafted the protocol and is the guarantor of the review. EW contributed to the search strategy and study design. All authors provided input on the study design and protocol development and contributed to the final manuscript.

Funding:

NW's research is funded by the Wellcome Trust as part of a Clinical PhD in Global Health at King's Centre for Global Health and Health Partnerships, London, UK. NS' research is supported by the National Institute for Health Research (NIHR) Collaboration for Leadership in Applied Health Research and Care South London at King's College Hospital NHS Foundation Trust. NS is also a member of King's Improvement Science, which is part of the NIHR CLAHRC South London and comprises a specialist team of improvement scientists and senior researchers based at King's College London. Its work is funded by King's Health Partners (Guy's and St Thomas' NHS Foundation Trust, King's College Hospital NHS Foundation Trust, King's College London and South London and Maudsley NHS Foundation Trust), Guy's and St Thomas' Charity, the Maudsley Charity and the Health Foundation. The funding bodies had no input to the content of this study or manuscript. The views expressed are those of the authors and not necessarily those of the Wellcome Trust, the NHS, the NIHR or the Department of Health and Social Care.

Competing interests:

The authors declare no competing interests.

Confidential: For Review Only

Improving outcomes for neonates with gastroschisis in low- and middle-income countries: a systematic review protocol

Supplementary File 1

Database(s): **Ovid MEDLINE(R) In-Process & Other Non-Indexed Citations and Ovid MEDLINE(R)** 1946 to Present

#	Searches	Results
1	exp Infant, Newborn/	564962
2	(neonate* or newborn*).mp.	723943
3	1 or 2	726361
4	anorectal malformations/	499
5	anus, imperforate/	2460
6	esophageal atresia/	3337
7	intestinal atresia/	1996
8	hernias, diaphragmatic, congenital/	4294
9	gastroschisis/	1128
10	duodenal obstruction/	3179
11	intestinal volvulus/	2298
12	Gastroschisis/	1128
13	Anorectal Malformations/	499
14	Anus, Imperforate/	2460
15	Duodenal Obstruction/	3179
16	Intestinal Atresia/	1996

17	Intestinal Volvulus/	2298
18	Hernias, Diaphragmatic, Congenital/	4294
19	Esophageal Atresia/	3337
20	Tracheoesophageal Fistula/	3467
21	("congenital anomal*" or "congenital abnormal*" or "birth defect*" or gastroschisis or exomphalos or omphaloc?ele or "abdominal wall defect" or "anorectal malformation" or "imperforate anus" or "duodenal atresia" or "colon* atresia" or "intestin* atresia" or malrotation or "congenital diaphragmatic hernia*" or "?esophageal atresia" or "tracheo-?esophageal fistula" or volvulus or "congenital malformation*" or "jejunal atresia" or "jejuno-ileal atresia" or "ileal atresia" or "Hirschsprung's disease" or aganglionosis or "apple peel syndrome*" or "anorectal stenos?s" or "anal atresia*").mp.	98434
22	or/4-21	104428
23	Developing Countries/	69831
24	exp Africa/	233166
25	exp Asia, Central/	6779
26	exp Transcaucasia/	3966
27	exp Central America/	14334
28	Afghanistan/	2857
29	Albania/	762
30	American Samoa/	159
31	Argentina/	13689
32	Bangladesh/	9034
33	Bhutan/	343
34	Bolivia/	2275
35	"Bosnia and Herzegovina"/	1847

36	Brazil/	76758
37	Bulgaria/	6069
38	Cambodia/	2835
39	China/	132950
40	Comoros/	260
41	Croatia/	6164
42	Cuba/	4750
43	"Democratic People's Republic of Korea"/	184
44	Dominica/	84
45	Ecuador/	3085
46	Fiji/	834
47	Grenada/	117
48	Guyana/	621
49	Haiti/	2834
50	India/	91150
51	Indonesia/	8786
52	Iran/	20716
53	Iraq/	4218
54	Jamaica/	3295
55	Jordan/	3484
56	Kosovo/	124
57	Lebanon/	3590
58	Laos/	1634

59	Madagascar/	3026
60	Malaysia/	12977
61	Mauritius/	510
62	Mexico/	33915
63	Micronesia/	1074
64	Moldova/	642
65	Mongolia/	1532
66	Montenegro/	159
67	Myanmar/	1892
68	Nepal/	6570
69	Pakistan/	14361
70	Papua New Guinea/	3214
71	Paraguay/	691
72	Peru/	7366
73	Philippines/	7578
74	"Republic of Belarus"/	1992
75	Romania/	9348
76	Russia/	36547
77	Saint Lucia/	64
78	"Saint Vincent and the Grenadines"/	46
79	Samoa/	295
80	Serbia/	2531
81	Sri Lanka/	5179

82	Suriname/	855
83	Syria/	1400
84	Thailand/	23569
85	Timor-Leste/	148
86	Turkey/	29917
87	Ukraine/	15218
88	Vanuatu/	321
89	Venezuela/	4626
90	Vietnam/	10611
91	Yemen/	1234
92	(afghanistan or albania or algeria or "american samoa" or angola or argentina or armenia or azerbaijan or bangladesh or belarus or belize or benin or bhutan or bolivia or bosnia or botswana or brazil or bulgaria or "burkina faso" or burundi or "cabo verde" or "cape verde" or cambodia or cameroon or "central africa* republic" or chad or china or columbia or comoros or "costa rica" or "cote d'ivoire" or croatia or cuba or "democratic people's republic of korea" or "democratic republic of the congo" or djibouti or dominica or "dominica republic" or drc or "east timor" or eritrea or ecuador or "el salvador" or egypt or ethiopia or fiji or gabon or gambia or gaza or republic georgia or ghana or grenada or guatemala or guinea or "guinea-bissau" or guyana or haiti or herzegovina or honduras or india or indonesia or iran or "ivory coast" or jamaica or jordan or kazakhstan or kenya or kiribati or kosovo or "kyrgyz republic" or "lao pdr" or laos).mp.	825783
93	(lebanon or lesotho or liberia or libya or macedonia or madagascar or malawi or malaysia or maldives or mali or "marshall islands" or mauritania or mauritius or mexico or micronesia or moldova or mongolia or montenegro or morocco or mozambique or myanmar or namibia or nauru or nepal or nicaragua or nigeria or "timor-leste" or iraq or niger or panama or pakistan or "papua new guinea" or paraguay or peru or philippines or romania or russia or "russian federation" or rwanda or ruanda or "saint lucia" or "saint vincent and the grenadines" or samoa or "sao tome and principe" or senegal or senegambia or serbia or "sierra leone" or "solomon islands" or somalia or somaliland or "south africa" or "sri lanka" or "st lucia" or "st vincent and the grenadines" or "sub-saharan africa" or sudan or suriname or syria or "syrian arab republic" or swaziland or tajikistan or tanzania or thailand or togo or tonga or tunisia or turkey or	531582

	turkmenistan or tuvalu or uganda or ukraine or uzbekistan or vanuatu or venezuela or vietnam or "west bank" or yemen or yugoslavia or zaire or zambia or zimbabwe).mp.	
94	(kabul or Porto-Novo or Hogbonou or Adjace or Cotonou or Kutonu or Ouagadougou or Ouaga or Bujumbura or Usumbura or Phnom Penh or Bangui or Bangi or N'Djamena or Ndjamena or Fort Lamy or Moroni or Kinshasa or Asmara or Asmera or Addis Ababa or Addis Abeba or Banjul or Bathurst or Conakry or Bissau or Port-au-Prince or Pyongyang or Monrovia or Antananarivo or Tananarive or Tana or Lilongwe or Bamako or Maputo or Lourenco Marques or Kathmandu or Niamey or Kigali or Freetown or Free-town or Mogadishu or Xamar or Hamar or Muqdisho or Maqadishu or Juba or Dodoma or Dar es Salaam or Lome or Kampala or Harare or Salisbury or Yerevan or Dhaka or Dacca or Thimphu or Thimbu or Sucre or Charcas or La Plata or Chuquisaca or La Paz or Praia or Yaounde or Jaunde or Brazzaville or Yamoussoukro or Cairo or Accra).mp.	24386
95	(Tegucigalpa or Tegus or New Delhi or Jakarta or Nairobi or South Tarawa or Tarawa Teinainano or Pristina or Prishtina or Bishkek or Pishpek or Frunze or Vientiane or Maseru or Nouakchott or Palikir or Chisinau or Kishinev or Rabat or Nay Pyi Taw or Naypyidaw or Nepranytau or Naypyitaw or Kyetpyay or Pyinmana or Kyatpyay or Pyinmana or Yangon or Rangoon or Managua or Abuja or Lagos or Islamabad or Port Moresby or Moresby or Pom Town or Manila or Apia or Dakar or Honiara or Jayawardenepura or Jayewardenepura or Khartoum or Mbabane or Embabane or Lobamba or Damascus or Dushanbe or Dyushambe or Stalinabad or Dili or Kyiv or Kiev or Tashkent or Toshkent or Port Vila or Hanoi or Ha Noi or Sana'a or Sanaa or Sana or Lusaka or Ulaanbaatar or Ulan-Bator or Luanda or Tbilisi or Amman).mp.	21747
96	(("under developed" or underdeveloped or low income or middle income or developing or less developed or "third world" or poor or LMI or LLMI or LAMI) adj1 (countr* or nation* or economy or economies)).mp.	128848
97	(("resource limited" or "low resource") adj1 (countr* or setting*)).mp.	7852
98	("less resourced communit*" or LMIC or LMICs).mp.	2416
99	or/23-98	1391627
100	3 and 22 and 99	2565
101	exp animals/ not humans/	4448581
102	100 not 101	2551

BMJ Paediatrics Open

Improving outcomes for neonates with gastroschisis in low- and middle-income countries: a systematic review protocol

Journal:	*BMJ Paediatrics Open*
Manuscript ID	bmjpo-2018-000392.R1
Article Type:	Protocol
Date Submitted by the Author:	21-Nov-2018
Complete List of Authors:	Wright, Naomi; King's Centre for Global Health and Health Partnerships<br>Langer, Monica; Northwestern University<br>Norman, Irena; GKT School of Medical Education<br>Akhbari, Melika; GKT School of Medical Education<br>Wafford, Q. Eileen; Northwestern University<br>Ade-Ajayi, Niyi; King's College Hospital, Department of Paediatric Surgery<br>Davies, Justine; King's Centre for Global Health and Health Partnerships<br>Poenaru, Dan ; McGill University<br>Sevdalis, Nick; King's College London<br>Leather, Andy; King's Centre for Global Health and Health Partnerships
Keywords:	Paediatric Surgery, Congenital Abnorm, Clinical Procedures, Intensive Care, Nursing Care

SCHOLARONE™
Manuscripts

Improving outcomes for neonates with gastroschisis in low- and middle-income countries: a systematic review protocol

Naomi J Wright[1], Monica Langer[2], Irena CF Norman[3], Melika Akhbari[3], Q. Eileen Wafford[4], Niyi Ade-Ajayi[5], Justine Davies[1], Dan Poenaru[6] , Nick Sevdalis[7], Andy Leather[1]

[1] King's Centre for Global Health and Health Partnerships, School of Population Health and Environmental Sciences, King's College London, UK
[2] Northwestern University, Chicago, USA
[3] GKT School of Medical Education, King's College London, UK
[4] Galter Health Sciences Library and Learning Center, Northwestern University, Chicago, USA
[5] Paediatric Surgery Department, King's College Hospital, London, UK
[6] McGill University, Montreal, Canada
[7] Centre for Implementation Science, King's College London, UK

Author email addresses:
naomiwright@doctors.org.uk, langerm7@hotmail.com, irena.norman@kcl.ac.uk, melika.akhbari@kcl.ac.uk, q-wafford@northwestern.edu, niyi.ade-ajayi@nhs.net, Justine.davies@kcl.ac.uk, nick.sevdalis@kcl.ac.uk, andy.leather@kcl.ac.uk

Corresponding author:
Miss Naomi Wright MBChB (Hons) BSc (Hons) MRCS DCH MSc
Paediatric Surgery Registrar and Wellcome Trust Clinical PhD Fellow in Global Health
King's Centre for Global Health and Health Partnerships, Suite 2.13, Weston Education Centre, Cutcombe Road, London, SE5 9RJ.

PROSPERO Registration: CRD42018095349
Registered 3rd July 2018
Title and abstract screening complete.
Full text screening and data extraction currently in progress.

Protocol prepared using PRISMA-P Guidelines

Confidential: For Review Only

ABSTRACT

Introduction: There is a significant disparity in outcomes for neonates with gastroschisis in high-income countries (HICs) compared to low- and middle-incomes countries (LMICs). Many LMICs report mortality rates between 75-100% compared to less than 4% in HICs.

Aim: To undertake a systematic review identifying postnatal interventions associated with improved outcomes for gastroschisis in LMICs.

Methods and analysis: Three search strings will be combined: 1) neonates; 2) gastroschisis and other gastrointestinal congenital anomalies requiring similar surgical care; 3) LMICs. Databases to be searched include: MEDLINE, EMBASE, Scopus, Wed of Science, ProQuest Dissertations and thesis global and the Cochrane Library. Grey literature will be identified through Open-Grey, ClinicalTrials.gov, WHO International Clinical Trials Registry and ISRCTN registry (Springer Nature). Additional studies will be sought from reference lists of included studies. Study screening, selection, data extraction and assessment of methodological quality will be undertaken by two reviewers independently and team consensus sought on discrepancies.

The primary outcome of interest is mortality. Secondary outcomes include: complications, requirement for ventilation, parenteral nutrition duration and length of hospital stay. Tertiary outcomes include service delivery and implementation outcomes. The methodology of the studies will be appraised. Descriptive statistics and outcomes will be summarised and discussed.

Ethics & dissemination: Ethical approval is not required since no new data is being collected. Dissemination will be via open access publication in a peer-reviewed medical journal and distribution amongst global health, global surgery and children's surgical collaborations and international conferences.

Conclusion: This study will systematically review literature focussed on postnatal interventions to improve outcomes from gastroschisis in LMICs. Findings can be used to help inform quality improvement projects in low-resource settings for patients with gastroschisis. In the first instance, results will be used to inform a Wellcome Trust funded multi-centre clinical interventional study aimed at improving outcomes for gastroschisis across sub-Saharan Africa.

Confidential: For Review Only

What is already known on this topic?

- Congenital anomalies are the 5th leading cause of death in children under 5-years of age globally.
- Gastroschisis is one of the commonest congenital anomalies with an increasing global incidence.
- Mortality due to gastroschisis is above 75% in many low- and middle-income countries (LMICs) compared to 4% in high-income countries.

What this study hopes to add:

- To systematically identify studies that evaluate postnatal interventions aimed at improving outcomes for neonates with gastroschisis in LMICs.
- To identify interventions used in the management of other congenital anomalies involving the gastrointestinal tract that are transferrable to the treatment of gastroschisis in LMICs.

Confidential: For Review Only

INTRODUCTION

Congenital anomalies are estimated to be the fifth leading cause of death in under 5-year olds globally (1). Gastroschisis (a condition where the intestines protrude through a hole in the abdominal wall at birth) is one of the commonest congenital anomalies and has been increasing in incidence globally (2-6). It occurs in approximately 1 in 2000 births. With an estimated 32 million births per year in sub-Saharan Africa (SSA) we would expect 16,000 neonates with gastroschisis to be born in the region annually. Indeed, Paediatric Surgeons across SSA report receiving between 1-15 cases per month (8). Since the 1960s mortality from gastroschisis has fallen in high-income countries (HICs) to less than 5% today (8). Mortality has fallen to a lesser extent in middle-income countries. Recent literature reports mortality rates of: 80%, Iran; 36%, Turkey; and 6-8%, Thailand (9-12). In low-income countries mortality remains high, with many SSA countries reporting mortality rates of 75 - 100% (7, 13-15).

Management of gastroschisis varies widely. The most common interventions in HICs are primary closure in the operating room or use of a preformed silo with gradual intestinal reduction and delayed closure, often at the cotside without general anaesthetic (16). Systematic reviews report comparable outcomes for both methods in HICs, but with lower ventilation requirements associated with the use of a preformed silo (17, 18). Preformed silo use has additional benefits for LMICs: it is low-technology; avoids neonatal anaesthesia and surgery; can be applied at the cotside by any trained healthcare personnel and reduces intensive care requirements due to lower intra-abdominal pressures (17). This is advantageous in low-resource settings where there is variable availability of paediatric

surgeons, deficient intensive care facilities, and safety of neonatal anaesthesia and surgery is limited by lack of trained staff and resources (19,20).

However, preformed silos are expensive and have been largely unavailable in LMICs and hence alternative strategies have been devised (12). Examples include use of an Alexis Wound Retractor as an alternative to the preformed silo, primary reduction at the cotside (Bianchi technique) and umbilical turban and flap closure (21-28). Furthermore, antenatal diagnosis, delivery in a tertiary paediatric surgery centre, pre-hospital management, neonatal resuscitation and nutrition are all fundamental components of care that impact survival (15). Interventions aimed at improving one or more of these components has the potential to significantly improve outcomes.

Some centres within low-resource settings have managed to achieve better survival from gastroschisis and other similar congenital anomalies involving the gastro-intestinal tract using one or more of the above interventions. However, to our knowledge, there has never been a systematic review to collate and analyse such evidence from LMIC settings. Hence, the focus of this systematic review is to identify postnatal pre-hospital and in-hospital interventions aimed at improving outcomes for neonates with gastroschisis in LMICs. This information is vital to inform quality improvement projects aimed at improving survival from gastroschisis in LMICs. In the first instance, the results of this review will be used in the design of a Wellcome Trust funded multi-centre clinical interventional study aimed at reducing mortality from gastroschisis in seven tertiary paediatric surgery centres in sub-Saharan Africa.

In this review, an 'intervention' is defined as any action taken to improve a patient's medical condition. This includes specific interventions for gastroschisis and generic interventions used for a wider range of congenital anomalies involving the gastrointestinal tract, which may also be beneficial for patients with gastroschisis. The review will not include antenatal interventions since another systematic review is currently in progress focussed on this topic.

METHODS AND ANALYSIS

Preferred Reporting Items for Systematic Review and Meta-Analysis Protocols (PRISMA-P) 2015 guidelines have been followed in this protocol (29). Supplementary file 1 details the PRISMA-P checklist and where each of the items is addressed in this protocol. If amendments to the protocol occur, they will be reported in the publication of the results.

Aim

To identify postnatal pre-hospital and in-hospital interventions associated with improved outcomes for neonates with gastroschisis in LMICs.

Objectives

1) To identify studies that evaluate postnatal interventions to improve mortality and morbidity for neonates with gastroschisis in LMICs.

Confidential: For Review Only

2) To identify generic surgical care interventions used in LMICs to manage neonates with a wider range of structural congenital anomalies involving the gastrointestinal tract, which may be transferrable to the care of neonates with gastroschisis.

3) To critically appraise the methodological quality of the evidence.

4) To provide an evidence-based summary of the condition-specific and generic neonatal surgical care interventions associated with improved outcomes for gastroschisis in LMICs to inform clinical practice and future studies.

Search strategy

A medical research librarian developed the search strategy in collaboration with members of the review team. The search was optimised by testing the sensitivity and specificity of the search terms during the development phase and revising the search strategy accordingly. The search strategy consists of controlled vocabulary and keywords for 1) the population - neonates, 2) the conditions - gastroschisis and a selection of structural congenital anomalies involving the gastrointestinal tract requiring a similar package of neonatal surgical care, and 3) the context - LMICs.

Neonates are defined as infants within the first 28-days of life. Terms for structural congenital anomalies involving the gastrointestinal tract are outlined in search string 2 (Table 1). These were derived from consensus amongst the authors according to what conditions may utilise similar neonatal surgical care as gastroschisis and thus have relevant transferrable interventions. The third search string includes all countries listed as low- or middle-income by the World Bank in 2018 and the varying terminology utilised to describe LMICs (30). Individual countries and major cities will be included.

The search strategy was developed in MEDLINE (Supplementary document 2). A highly sensitive search will be undertaken by employing truncation and wildcards and applying the Unqualified Searches (MP) tag to search text words. The search strategy will be adapted to MEDLINE (Ovid), EMBASE (Elsevier), Scopus (Elsevier), Web of Science (Clarivate Analytics), and the Cochrane Library. Each database will be searched from the date of inception. The search will not be restricted based on language or study design. Only human studies will be included. Literature reviews and reference lists of included studies will be searched for further studies suitable for inclusion.

Grey literature will be included to help mitigate the risks of publication bias and to identify the latest progress in the field. We will identify unpublished studies by searching the following grey literature sources: Open-Grey, ClinicalTrials.gov, WHO International Clinical Trials Registry and ISRCTN registry (Springer Nature). These were selected since they are major sources of grey literature for the biosciences providing comprehensive coverage. The following publication types will be included: dissertations/ theses, books/ book chapters, conference abstracts, editorials/ letters/ comments, newspapers/ trade journals, literature reviews, and research in progress. Experts in the field will be contacted to identify any

Confidential: For Review Only

ongoing research on this subject, which has yet to be published. The authors of identified grey literature will be contacted for a full report of data and findings where available.

Table 1: Three search strings to be utilised to identify studies to be included in the systematic review

Search String 1	Search String 2	Search String 3
newborn<br>neonate	congenital anomalies, congenital abnormalities, congenital malformation, birth defects, gastroschisis, exomphalos, omphalocele, abdominal wall defect, intestinal atresia, apple peel syndrome, duodenal atresia, duodenal obstruction, duodenal web, jejunal atresia, jejuno-ileal atresia, ileal atresia, colonic atresia, anorectal malformation, anorectal stenosis, imperforate anus, anal atresia, malrotation, volvulus, congenital diaphragmatic hernia, oesophageal atresia, tracheo-oesophageal fistula, Hirschsprung's Disease, Aganglionosis	low income countries,<br>middle income countries,<br>LMICs, LAMI, LMI<br>low resource settings, resource limited setting, less resourced communities,<br>developing countries, underdeveloped countries, third world countries, developing nations, low income nation,<br>sub-Saharan Africa,<br>Afghanistan, Albania, Algeria, American Samoa, Angola, Argentina, Armenia, Azerbaijan, Bangladesh, Belarus, Belize, Benin, Bhutan, Bolivia, Bosnia and Herzegovina, Botswana, Brazil, Bulgaria, Burkina Faso, Burundi, Cabo Verde, Cambodia, Cameroon, Central African Republic, Chad, China, Columbia, Comoros, Democratic Republic of the Congo, DRC, Republic of the Congo, Costa Rica, Cote d'Ivoire, Ivory Coast, Croatia, Cuba, Djibouti, Dominica, Ecuador, Egypt, El Salvador, Equatorial Guinea, Eritrea, Ethiopia, Fiji, Gabon, Gambia, Georgia, Ghana, Grenada, Guatemala, Guinea, Guinea-Bissau, Guyana, Haiti, Honduras, India, Indonesia, Islamic Republic of Iran, Iraq, Jamaica, Jordan, Kazakhstan, Kenya, Kiribati, Democratic People's Republic of Korea, Kosovo, Kyrgyz Republic, Lao PDR, Laos, Lebanon, Lesotho, Liberia, Libya, Macedonia Republic, Madagascar, Malawi, Malaysia, Maldives, Mali, Marshall Islands, Mauritania, Mauritius, Mexico, Micronesia, Moldova, Mongolia, Montenegro, Morocco, Mozambique, Myanmar, Namibia, Nauru, Nepal, Nicaragua, Niger, Nigeria, Pakistan, Panama, Papua New Guinea, Paraguay, Peru, Philippines, Romania, Russian Federation, Rwanda, Samoa, Sao Tome and Principe, Senegal, Serbia, Sierra Leone, Solomon Islands, Somalia, Somaliland, South Africa, Sri Lanka, St. Lucia, Saint Lucia, St. Vincent and Grenadines, Saint Vincent and the Grenadines, Sudan, Suriname, Swaziland, Syrian Arab Republic, Syria, Tajikistan, Tanzania, Thailand, Timor-Leste, East Timor, Togo, Tonga, Tunisia, Turkey, Turkmenistan, Tuvalu, Uganda, Ukraine, Uzbekistan, Vanuatu, Venezuela, Vietnam, West Bank and Gaza, Republic of Yemen, Zambia, Zimbabwe.<br>Kabul, Porto-Novo, Hogbonou, Adjace, Cotonou, Kutonu, Ouagadougou, Ouaga, Bujumbura, Usumbura, Phnom Penh, Bangui, Bangi, N'Djamena, Ndjamena, Fort Lamy, Moroni, Kinshasa, Asmara, Asmera, Addis Ababa, Addis Abeba, Banjul, Bathurst, Conakry, Bissau, Port-au-Prince, Pyongyang, Monrovia, Antananarivo, Tananarive, Tana, Lilongwe, Bamako, Maputo, Lourenco Marques, Kathmandu, Niamey, Kigali, Freetown, Free-town, Mogadishu, Xamar, Hamar, Muqdisho, Maqadishu, Juba, Dodoma, Dar es Salaam, Lome, Kampala, Harare, Salisbury, Yerevan, Dhaka, Dacca, Thimphu, Thimbu, Sucre, Charcas, La Plata, Chuquisaca, La Paz, Praia, Yaounde, Jaunde, Brazzaville, Yamoussoukro, Cairo, Accra, Tegucigalpa, Tegus, New Delhi, Jakarta, Nairobi, South Tarawa, Tarawa Teinainano, Pristina, Prishtina, Bishkek, Pishpek, Frunze, Vientiane, Maseru, Nouakchott, Palikir, Chisinau, Kishinev, Rabat, Nay Pyi Taw, Naypyidaw, Nepranytau, Naypyitaw, Kyetpyay, Pyinmana, Kyatpyay, Pyinmana, Yangon, Rangoon, Managua, Abuja, Lagos, Islamabad, Port Moresby, Moresby, Pom Town, Manila, Apia, Dakar, Honiara, Jayawardenepura, Jayewardenepura, Khartoum, Mbabane, Embabane, Lobamba, Damascus, Dushanbe, Dyushambe, Stalinabad, Dili, Kyiv, Kiev, Tashkent, Toshkent, Port Vila, Hanoi, Ha Noi, Sana'a, Sanaa, Sana, Lusaka, Ulaanbaatar, Ulan-Bator, Luanda, Tbilisi, Amman

Confidential: For Review Only

Inclusion/ exclusion criteria

Conditions

Conditions to be included are listed in column 2 of Table 1 (search string 2). Neonates with these structural congenital anomalies involving the gastrointestinal tract commonly present with a life-threatening emergency requiring a similar package of care within the neonatal period.

Structural congenital anomalies involving the gastrointestinal tract to be excluded from the search criteria include: biliary atresia, choledochal cyst and all other conditions not listed under search string 2. These conditions often present outside of the neonatal period.

Setting and participants

Studies containing preterm and term neonates presenting within the first 28-days of life with gastroschisis or one of the structural congenital anomalies listed in search string 2 will be included. Studies including just patients who have previously received care and re-presented with a complication or need for further intervention will be excluded. Only studies which have been undertaken in LMICs will be included.

Interventions

All pre-hospital and in-hospital postnatal interventions for the care of neonates with gastroschisis in LMICs will be included. Generic interventions related to the care of neonates with a structural congenital anomaly involving the gastrointestinal tract will be included. Antenatal interventions will be excluded because they are currently being evaluated in a separate systematic review.

Types of interventions will be categorised into specific interventions for neonates with gastroschisis and generic neonatal surgical care interventions for structural congenital anomalies involving the gastrointestinal tract. Generic interventions will be sub-categorised into: pre-hospital care and transportation, place of delivery, neonatal resuscitation and care, staffing, access to parenteral nutrition and other. Operative interventions related specifically and solely to a condition other than gastroschisis will be excluded. For example, operative techniques for oesophageal atresia or anorectal malformation.

Outcome measures

The primary outcome of the review will be mortality. This will include: all-cause in-hospital mortality, mortality within the neonatal period (within 28-days of life) and 30-day post-intervention mortality. Secondary outcomes will include: complications (post-primary intervention*), requirement for ventilation (yes/ no, number of days), duration of parenteral nutrition (days) and length of hospital stay (days). Complications will be determined using the Clavien-Dindo Classification, Table 2 (31).

Confidential: For Review Only

*Primary intervention is defined as the first intervention the neonate received for bowel coverage, including application of a preformed silo at the cot-side.

An improved primary or secondary outcome will be defined as a significant difference with a p value < 0.05.

Table 2: Clavien-Dindo Classification of Complications (31)

Grade	Definition
I	Any deviation from the normal post-operative course without the need for pharmacological treatment or surgical, endoscopic, and radiological interventions. Allowed therapeutic regimes are as follows: drugs as anti-emetics, anti-pyretics, analgesics, diuretics, electrolytes, parenteral nutrition, and physiotherapy. This grade also includes wound infections opened at the bedside.
II	Requiring pharmacological treatment with drugs other than such allowed for grade I complications. Blood transfusions are also included.
III	Requiring surgical, endoscopic, or radiological intervention.
IIIa	Intervention not under general anaesthesia.
IIIb	Intervention under general anaesthesia.
IV	Life-threatening complication requiring ICU management.
IVa	Single organ dysfunction (including dialysis).
IVb	Multi-organ dysfunction.
V	Death of a patient.

Tertiary outcomes include service delivery and implementation outcomes (Table 3). Implementation strategies will also be analysed. An implementation strategy is defined as the method(s) or technique(s) used to enhance the adoption, implementation, and sustainability of a clinical program or practice (32).

Table 3: Definition of Implementation Outcomes (33)

Implementation outcome	Definition
Acceptability	Perception amongst stakeholders that the new intervention is agreeable.
Adoption	Intention to apply new intervention
Appropriateness	Perceived relevance of the intervention for the setting and problem
Feasibility	Extent to which an intervention can be applied
Fidelity	The proportion of management protocol components completed as intended.
Coverage	The proportion of eligible patients who actually receive the intervention.
Cost	Costs of the intervention, including the delivery strategy.
Sustainability	Extent to which a new intervention becomes routinely available/ is maintained post-introduction.

Study screening

References identified through the electronic search engines will be entered into Covidence and duplicates removed (34). Two reviewers will independently screen the titles and abstracts of all references. All potentially relevant articles will have the full text reviewed in

https://mc.manuscriptcentral.com/bmjpo
Confidential: For Review Only

duplicate against the eligibility criteria. This will include the full text of articles in languages other than English, which will be translated. Inter-rater reliability will be assessed following the screening of the first 50 abstracts through a review of the decisions made and discussion amongst the wider authorship. Consensus will be sought on the hierarchy of reasons for rejecting studies to ensure consistency amongst the study team. Any discrepancies during the screening process will be highlighted in Covidence and will be resolved by consensus with the wider authorship group. All reviewers are trained in systematic review methods. The search results will be represented using a PRISMA flow chart (35).

Data extraction

Data will be extracted in duplicate by two reviewers and entered into a pre-determined data collection form. Data will be collected on the study type, country, year of publication, journal of publication, authors' names, number of patients, patient demographics (including proportion with simple and complex gastroschisis*, gestational age, weight, time from birth to presentation at the study hospital and American Association of Anesthesiologists Score at the time of primary intervention), pre-hospital and in-hospital intervention(s), implementation strategy where relevant, primary and secondary clinical outcomes as detailed above, service delivery outcomes and implementation outcomes if available. The two data extraction databases will be compared and any discrepancies discussed with the wider authorship to determine consensus. The data collection form will be sent to investigators of unpublished studies to obtain such data where available.

*Patients with bowel necrosis, perforation, atresia or closing/ closed gastroschisis.

Data synthesis

Descriptive statistics will be used to present the interventions and outcomes in results tables, accompanied by a narrative synthesis. Interventions will be categorised into gastroschisis-specific and generic neonatal surgical care, with the latter being further sub-categorised as detailed above. Because a wide range of interventions (often a group of interventions combined) and outcomes will be evaluated, it is unlikely that a meta-analysis will be feasible. However, if there is appropriate data, a meta-analysis will be undertaken. Appropriate data will be defined as: two or more studies comparing the mortality between two or more of the same interventions so we can pool the data and perform a meta-analysis. For example two or more studies comparing the mortality outcome between intervention 'a' with intervention 'b'. Meta-analysis will be undertaken in Stata and results presented using a forest plot. If there are over 10 studies in the meta-analysis a funnel plot will be undertaken to assess publication bias and a Galbraith plot to investigate heterogeneity in effect sizes. The quality of evidence will be assessed following GRADE guidelines (36).

Methodological quality appraisal and bias assessment

Confidential: For Review Only

The methodological quality of the individual studies will be assessed and the findings summarised in a table to aid interpretation. This will be incorporated into the narrative synthesis. Cochrane Risk of Bias for Non-Randomised Studies of Interventions (ROBINS-I) and the revised tool for Risk of Bias in randomised trials (RoB 2.0) will be used to assess quantitative studies (37, 38). This will be undertaken by two reviewers independently and team consensus sought for discrepancies.

DISCUSSION

To our knowledge, this will be the first systematic review focussed on postnatal interventions to improve outcomes from gastroschisis in LMICs. Such a review is vital to address the current outcome disparities, with many neonates with gastroschisis dying in LMICs and the majority surviving in HICs (12, 15). It is hoped lessons learned in centres with better outcomes within LMICs can be evaluated and shared amongst the global community to improve outcomes and inform future interventional studies. A wider range of congenital anomalies involving the gastro-intestinal tract will be incorporated into the study to help identify generic neonatal surgical care interventions that have the potential to also improve outcomes for neonates with gastroschisis. This information may also help to inform clinical practice for a wider range of structural congenital anomalies involving the gastrointestinal tract. The systematic review may also highlight areas for improvement in HICs, such as cost reduction.

Strengths and limitations

This study is unique in its focus and comprehensive search strategy incorporating both original articles, grey literature, published and unpublished work. Incorporating a wider range of structural congenital anomalies within the search strategy will help to identify neonatal surgical care interventions utilised in LMICs that could be beneficial for patients with gastroschisis. Identified articles in languages other than English will be translated so they can be included within the review.

Although the search strategy has been designed to be optimally inclusive, it is possible that articles could be missed. The initial search will be undertaken in English and some articles without English translation of the title or abstract could be missed. This systematic review will only include studies undertaken within LMICs; it may be possible that low-technology interventions utilised within HICs could also benefit gastroschisis care in low-resource settings.

Ethics and dissemination

This systematic review will analyse previously published historical data, thus does not require ethical approval.

The results will be submitted for open access publication in a peer-reviewed medical journal. The publication will be disseminated amongst the PaedSurg Africa Research Collaboration and Global PaedSurg Research Collaboration consisting of hundreds of children's surgical care providers across the globe (39). It will also be shared amongst

Confidential: For Review Only

members of the Global Initiative for Children's Surgery (GICS), which includes all members of the multi-disciplinary team caring for neonates with gastroschisis and international organisations, policy makers and representatives from the World Health Organisation (40). Results will be disseminated using social media. Findings will be presented internationally with a focus on global health, global surgery, paediatric and paediatric surgical conferences in LMICs.

The results will help to inform the development of an interventional care bundle to be evaluated in a Wellcome Trust funded multi-centre interventional study aimed at improving survival in neonates with gastroschisis in LMICs. This will be undertaken in seven tertiary paediatric surgery centres across sub-Saharan Africa between 2018 - 2020.

Acknowledgements

We thank the library services at King's College London, UK; Northwestern University, Chicago, USA; and Elena Guadagno, MLIS, and Alex Amar, MLIS, at McGill University Health Centre (MUHC), Montreal, Canada for their help with the literature search.

Confidential: For Review Only

Confidential: For Review Only

REFERENCES

1. Global Burden of Disease (GBD) Child Mortality Collaborators. Global, regional, national, and selected subnational levels of stillbirths, neonatal, infant, and under-5 mortality, 1980-2015: a systematic analysis for the Global Burden of Disease Study 2015. *Lancet* 2016;388(10053):1725-74.
2. Agugua NE, Nwako FA. Gastroschisis a fifteen-year experience. *West Afr J Med* 1990;9(2):147-50.
3. Alvarez SM, Burd RS. Increasing prevalence of gastroschisis repairs in the United States: 1996-2003. *J Pediatr Surg* 2007;42(6):943-6.
4. Arnold M. Is the incidence of gastroschisis rising in South Africa in accordance with international trends? A retrospective analysis at Pretoria Academic and Kalafong Hospitals, 1981-2001. *S Afr J Surg* 2004;42(3):86-8.
5. Benjamin BG, Ethen MK, Van Hook CL, et al. Gastroschisis prevalence in Texas 1999-2003. *Birth Defects Res A Clin Mol Teratol* 2010;88(3):178-85.
6. Tan KB, Tan KH, Chew SK et al. Gastroschisis and omphalocele in Singapore: a ten-year series from 1993 to 2002. *Singapore Med J* 2008;49(1):31-6.
7. Wright NJ, Zani A, Ade-Ajayi N. Epidemiology, management and outcome of gastroschisis in Sub-Saharan Africa: Results of an international survey. *Afr J Paediatr Surg* 2015;12(1):1-6.
8. Bradnock TJ, Marven S, Owen A et al. Gastroschisis: one year outcomes from national cohort study. *BMJ* 2011;343:d6749.
9. Askarpour S, Ostadian N, Javaherizadeh H et al. Omphalocele, gastroschisis: epidemiology, survival, and mortality in Imam Khomeini hospital, Ahvaz-Iran. *Pol Przegl Chir* 2012;84(2):82-5.
10. Erdogan D, Azili MN, Cavusoglu YH et al. 11-year experience with gastroschisis: factors affecting mortality and morbidity. *Iran J Pediatr* 2012;22(3):339-43.
11. Niramis R, Suttiwongsing A, Buranakitjaroen V et al. Clinical outcome of patients with gastroschisis: what are the differences from the past? *J Med Assoc Thai* 2011;94 Suppl 3:S49-56.
12. Sangkhathat S, Patrapinyokul S, Chiengkriwate P et al. Infectious complications in infants with gastroschisis: an 11-year review from a referral hospital in southern Thailand. *J Pediatr Surg* 2008;43(3):473-8.
13. Wesonga AS, Fitzgerald TN, Kabuye R et al. Gastroschisis in Uganda: Opportunities for improved survival. *J Pediatr Surg* 2016;51(11):1772-7.
14. Apfeld JC, Wren SM, Macheka N et al. Infant, maternal, and geographic factors influencing gastroschisis related mortality in Zimbabwe. *Surgery* 2015;158(6):1475-80.
15. Ford K, Poenaru D, Moulot O et al. Gastroschisis: Bellwether for neonatal surgery capacity in low resource settings? *J Pediatr Surg* 2016;51(8):1262-7.
16. Zani A, Ruttenstock E, Davenport M et al. Is there unity in Europe? First survey of EUPSA delegates on the management of gastroschisis. *Eur J Pediatr Surg* 2013;23(1):19-24.
17. Ross AR, Eaton S, Zani A et al. The role of preformed silos in the management of infants with gastroschisis: a systematic review and meta-analysis. *Pediatr Surg Int* 2015;31(5):473-83.
18. Pastor AC, Phillips JD, Fenton SJ et al. Routine use of a SILASTIC spring-loaded silo for infants with gastroschisis: a multicentre randomized controlled trial. *J Pediatr Surg* 2008;43:1807-12.

19. Bosenberg AT. Neonatal anesthesia with limited resources. *Paediatr Anaesth* 2014;24(1):98-105.
20. Krishnaswami S, Nwomeh BC, Ameh AE. The pediatric surgery workforce in low- and middle-income countries: problems and priorities. *Semin Pediatr Surg* 2016;25(1):32-42.
21. Emami CN, Youssef F, Baird RJ et al. A risk-stratified comparison of fascial versus flap closure techniques on the early outcomes of infants with gastroschisis. *J Pediatr Surg* 2015;50(1):102-6.
22. Duncan ND, Brown B, Dundas SE et al. "Minimal intervention management" for gastroschisis: a preliminary report. *West Indian Med J* 2005;54(2):152-4.
23. Ferreira C, G., Lacreuse I, Geslin D et al. Staged gastroschisis closure using Alexis wound retractor: first experiences. *Pediatr Surg Int* 2014;30:305-11.
24. Sandler A, Lawrence J, Meehan J. A “plastic” sutureless abdominal wall closure in gastroschisis. *J Pediatr Surg* 2004;39(5):738-41.
25. Kanamori Y. Operative general surgery in neonates and infants. Gastroschisis and Omphalocele. Taguchi T, editor: Springer Japan; 2016.
26. Kusafuka J, Yamataka A, Okazaki T et al. Gastroschisis reduction using ‘Applied Alexis”, a wound protector and retractor. *Pediatr Surg Int* 2005;21:925-7.
27. Ogasawara Y, Okazaki T, Kato Y et al. Spontaneous sutureless closire of the abdominal wall defect in gastroschisis using a commercial wound retractor system. *Pediatr Surg Int* 2009;25:973-6.
28. Bisaliev BN, Tsap NA. [Experience of Treatment of Newborn Children with Gastroschisis]. *Vestn Khir Im I I Grek* 2015;174(6):46-51.
29. Shamseer L, Moher D, Clarke M et al. Preferred reporting items for systematic review and meta-analysis protocols (PRISMA-P) 2015: elaboration and explanation. *BMJ* 2015;350:g7647.
30. World Bank. World Bank Country and Lending Groups. 2018. https://datahelpdesk.worldbank.org/knowledgebase/articles/906519-world-bank-country-and-lending-groups (Accessed 16th April 2018).
31. Clavien PA, Barkun J, de Oliveira ML et al. The Clavien-Dindo classification of surgical complications: five-year experience. *Ann Surg* 2009;250(2):187-96.
32. Proctor EK, Powell BJ, McMillen JC. Implementation strategies: recommendations for specifying and reporting. *Implement Sci* 2013;8:139.
33. Proctor E, Silmere H, Raghavan R et al. O
utcomes for Implementation Research: Conceptual Distinctions, Measurement Challenges, and Research Agenda. *Adm Policy Ment Health* 2011;38:65-76.
34. Cochrane. Covidence. www.covidence.org (Accessed 16th April 2018).
35. Moher D, Liberati A, Tetzlaff J et al. Preferred reporting items for systematic reviews and meta-analyses: the PRISMA statement. *J Clin Epidemiol* 2009;62(10):1006-12.
36. Balshem H, Helfand M, Schunemann HJ, Oxman AD, Kunz R, Brozek J, et al. GRADE guidelines: 3. Rating the quality of evidence. J Clin Epidemiol. 2011;64(4):401-6.
37. Higgins J, Sterne J, Savovic J et al. A revised tool for assessing risk of bias in randomized trials. *Cochrane Database Syst Rev* 2016;10(Supplement 1).
38. Sterne JA, Hernan MA, Reeves BC et al. ROBINS-I: a tool for assessing risk of bias in non-randomised studies of interventions. *BMJ* 2016;355:i4919.
39. Global PaedSurg Research Collaboration. www.globalpaedsurg.com (Accessed 16th April 2018).

Confidential: For Review Only

40. Goodman LF, St-Louis E, Yousef Y et al. The Global Initiative for Children's Surgery: Optimal Resources for Improving Care. *Eur J Pediatr Surg* 2017.

Confidential: For Review Only

Contributions:

NW and ML conceived the idea for the study and designed the protocol. NW drafted the protocol, designed the search strategy in conjunction with EW and is the guarantor of the review. EW contributed to the design of the search strategy and undertook the search. All authors provided input on the study design and protocol development and contributed to the final manuscript.

Funding:

NW's research is funded by the Wellcome Trust as part of a Clinical PhD in Global Health at King's Centre for Global Health and Health Partnerships, London, UK. NS' research is supported by the National Institute for Health Research (NIHR) Collaboration for Leadership in Applied Health Research and Care South London at King's College Hospital NHS Foundation Trust. NS is also a member of King's Improvement Science, which is part of the NIHR CLAHRC South London and comprises a specialist team of improvement scientists and senior researchers based at King's College London. Its work is funded by King's Health Partners (Guy's and St Thomas' NHS Foundation Trust, King's College Hospital NHS Foundation Trust, King's College London and South London and Maudsley NHS Foundation Trust), Guy's and St Thomas' Charity, the Maudsley Charity and the Health Foundation. The funding bodies had no input to the content of this study or manuscript. The views expressed are those of the authors and not necessarily those of the Wellcome Trust, the NHS, the NIHR or the Department of Health and Social Care.

Competing interests:

The authors declare no competing interests.

Confidential: For Review Only

Supplementary File 1

Improving outcomes for neonates with gastroschisis in low- and middle-income countries: a systematic review protocol

PRISMA-P (Preferred Reporting Items for Systematic review and Meta-Analysis Protocols) 2015 checklist: recommended items to address in a systematic review protocol with details of where each item is addressed in this protocol

Confidential: For Review Only

Section and topic	Item No	Checklist item	Section where each item is addressed in the protocol
**ADMINISTRATIVE INFORMATION**			
Title:			
Identification	1a	Identify the report as a protocol of a systematic review	Title page
Update	1b	If the protocol is for an update of a previous systematic review, identify as such	N/A
Registration	2	If registered, provide the name of the registry (such as PROSPERO) and registration number	Title page
Authors:			
Contact	3a	Provide name, institutional affiliation, e-mail address of all protocol authors; provide physical mailing address of corresponding author	Title page
Contributions	3b	Describe contributions of protocol authors and identify the guarantor of the review	Contributions
Amendments	4	If the protocol represents an amendment of a previously completed or published protocol, identify as such and list changes; otherwise, state plan for documenting important protocol amendments	Methods and Analysis
Support:			
Sources	5a	Indicate sources of financial or other support for the review	Funding
Sponsor	5b	Provide name for the review funder and/or sponsor	Funding
Role of sponsor or funder	5c	Describe roles of funder(s), sponsor(s), and/or institution(s), if any, in developing the protocol	Funding
**INTRODUCTION**			
Rationale	6	Describe the rationale for the review in the context of what is already known	Introduction
Objectives	7	Provide an explicit statement of the question(s) the review will address with reference to participants, interventions, comparators, and outcomes (PICO)	Aim and Objectives
**METHODS**			

Eligibility criteria	8	Specify the study characteristics (such as PICO, study design, setting, time frame) and report characteristics (such as years considered, language, publication status) to be used as criteria for eligibility for the review	Search strategy, inclusion/ exclusion criteria
Information sources	9	Describe all intended information sources (such as electronic databases, contact with study authors, trial registers or other grey literature sources) with planned dates of coverage	Search strategy
Search strategy	10	Present draft of search strategy to be used for at least one electronic database, including planned limits, such that it could be repeated	Table 1 and Supplementary Document 2
Study records:			
Data management	11a	Describe the mechanism(s) that will be used to manage records and data throughout the review	Study screening and data extraction
Selection process	11b	State the process that will be used for selecting studies (such as two independent reviewers) through each phase of the review (that is, screening, eligibility and inclusion in meta-analysis)	Study screening
Data collection process	11c	Describe planned method of extracting data from reports (such as piloting forms, done independently, in duplicate), any processes for obtaining and confirming data from investigators	Data extraction
Data items	12	List and define all variables for which data will be sought (such as PICO items, funding sources), any pre-planned data assumptions and simplifications	Data extraction
Outcomes and prioritization	13	List and define all outcomes for which data will be sought, including prioritization of main and additional outcomes, with rationale	Outcome measures
Risk of bias in individual studies	14	Describe anticipated methods for assessing risk of bias of individual studies, including whether this will be done at the outcome or study level, or both; state how this information will be used in data synthesis	Methodological quality appraisal and bias assessment
Data synthesis	15a	Describe criteria under which study data will be quantitatively synthesised	Data synthesis
	15b	If data are appropriate for quantitative synthesis, describe planned summary measures, methods of handling data and methods of combining data from studies, including any planned exploration of consistency (such as I^2, Kendall's τ)	Data synthesis
	15c	Describe any proposed additional analyses (such as sensitivity or subgroup analyses, meta-regression)	N/A
	15d	If quantitative synthesis is not appropriate, describe the type of summary planned	Data synthesis
Meta-bias(es)	16	Specify any planned assessment of meta-bias(es) (such as publication bias across studies, selective reporting within studies)	Data synthesis
Confidence in cumulative evidence	17	Describe how the strength of the body of evidence will be assessed (such as GRADE)	Data synthesis

*** It is strongly recommended that this checklist be read in conjunction with the PRISMA-P Explanation and Elaboration (cite when available) for important clarification on the items. Amendments to a review protocol should be tracked and dated. The copyright for PRISMA-P (including checklist) is held by the PRISMA-P Group and is distributed under a Creative Commons Attribution Licence 4.0.**

From: Shamseer L, Moher D, Clarke M, Ghersi D, Liberati A, Petticrew M, Shekelle P, Stewart L, PRISMA-P Group. Preferred reporting items for systematic review and meta-analysis protocols (PRISMA-P) 2015: elaboration and explanation. BMJ. 2015 Jan 2;349(jan02 1):g7647.

Confidential: For Review Only

Supplementary File 2

Improving outcomes for neonates with gastroschisis in low- and middle-income countries: a systematic review protocol

Database(s): **Ovid MEDLINE(R) In-Process & Other Non-Indexed Citations and Ovid MEDLINE(R)** 1946 to Present

#	Searches	Results
1	exp Infant, Newborn/	564962
2	(neonate* or newborn*).mp.	723943
3	1 or 2	726361
4	anorectal malformations/	499
5	anus, imperforate/	2460
6	esophageal atresia/	3337
7	intestinal atresia/	1996
8	hernias, diaphragmatic, congenital/	4294
9	gastroschisis/	1128
10	duodenal obstruction/	3179
11	intestinal volvulus/	2298
12	Gastroschisis/	1128
13	Anorectal Malformations/	499
14	Anus, Imperforate/	2460
15	Duodenal Obstruction/	3179
16	Intestinal Atresia/	1996
17	Intestinal Volvulus/	2298
18	Hernias, Diaphragmatic, Congenital/	4294
19	Esophageal Atresia/	3337
20	Tracheoesophageal Fistula/	3467
21	("congenital anomal*" or "congenital abnormal*" or "birth defect*" or gastroschisis or exomphalos or omphaloc?ele or "abdominal wall defect" or "anorectal malformation" or "imperforate anus" or "duodenal atresia" or "colon* atresia" or "intestin* atresia" or malrotation or "congenital diaphragmatic hernia*" or "?esophageal atresia" or "tracheo-?esophageal fistula" or volvulus or "congenital malformation*" or "jejunal atresia" or "jejuno-ileal atresia" or "ileal atresia" or "Hirschsprung's disease" or aganglionosis or "apple peel syndrome*" or "anorectal stenos?s" or "anal atresia*").mp.	98434
22	or/4-21	104428
23	Developing Countries/	69831

24	exp Africa/	233166
25	exp Asia, Central/	6779
26	exp Transcaucasia/	3966
27	exp Central America/	14334
28	Afghanistan/	2857
29	Albania/	762
30	American Samoa/	159
31	Argentina/	13689
32	Bangladesh/	9034
33	Bhutan/	343
34	Bolivia/	2275
35	"Bosnia and Herzegovina"/	1847
36	Brazil/	76758
37	Bulgaria/	6069
38	Cambodia/	2835
39	China/	132950
40	Comoros/	260
41	Croatia/	6164
42	Cuba/	4750
43	"Democratic People's Republic of Korea"/	184
44	Dominica/	84
45	Ecuador/	3085
46	Fiji/	834
47	Grenada/	117
48	Guyana/	621
49	Haiti/	2834
50	India/	91150
51	Indonesia/	8786
52	Iran/	20716
53	Iraq/	4218
54	Jamaica/	3295
55	Jordan/	3484

56	Kosovo/	124
57	Lebanon/	3590
58	Laos/	1634
59	Madagascar/	3026
60	Malaysia/	12977
61	Mauritius/	510
62	Mexico/	33915
63	Micronesia/	1074
64	Moldova/	642
65	Mongolia/	1532
66	Montenegro/	159
67	Myanmar/	1892
68	Nepal/	6570
69	Pakistan/	14361
70	Papua New Guinea/	3214
71	Paraguay/	691
72	Peru/	7366
73	Philippines/	7578
74	"Republic of Belarus"/	1992
75	Romania/	9348
76	Russia/	36547
77	Saint Lucia/	64
78	"Saint Vincent and the Grenadines"/	46
79	Samoa/	295
80	Serbia/	2531
81	Sri Lanka/	5179
82	Suriname/	855
83	Syria/	1400
84	Thailand/	23569
85	Timor-Leste/	148
86	Turkey/	29917
87	Ukraine/	15218

88	Vanuatu/	321
89	Venezuela/	4626
90	Vietnam/	10611
91	Yemen/	1234
92	(afghanistan or albania or algeria or "american samoa" or angola or argentina or armenia or azerbaijan or bangladesh or belarus or belize or benin or bhutan or bolivia or bosnia or botswana or brazil or bulgaria or "burkina faso" or burundi or "cabo verde" or "cape verde" or cambodia or cameroon or "central africa* republic" or chad or china or columbia or comoros or "costa rica" or "cote d'ivoire" or croatia or cuba or "democratic people's republic of korea" or "democratic republic of the congo" or djibouti or dominica or "dominica republic" or drc or "east timor" or eritrea or ecuador or "el salvador" or egypt or ethiopia or fiji or gabon or gambia or gaza or republic georgia or ghana or grenada or guatemala or guinea or "guinea-bissau" or guyana or haiti or herzegovina or honduras or india or indonesia or iran or "ivory coast" or jamaica or jordan or kazakhstan or kenya or kiribati or kosovo or "kyrgyz republic" or "lao pdr" or laos).mp.	825783
93	(lebanon or lesotho or liberia or libya or macedonia or madagascar or malawi or malaysia or maldives or mali or "marshall islands" or mauritania or mauritius or mexico or micronesia or moldova or mongolia or montenegro or morocco or mozambique or myanmar or namibia or nauru or nepal or nicaragua or nigeria or "timor-leste" or iraq or niger or panama or pakistan or "papua new guinea" or paraguay or peru or philippines or romania or russia or "russian federation" or rwanda or ruanda or "saint lucia" or "saint vincent and the grenadines" or samoa or "sao tome and principe" or senegal or senegambia or serbia or "sierra leone" or "solomon islands" or somalia or somaliland or "south africa" or "sri lanka" or "st lucia" or "st vincent and the grenadines" or "sub-saharan africa" or sudan or suriname or syria or "syrian arab republic" or swaziland or tajikistan or tanzania or thailand or togo or tonga or tunisia or turkey or turkmenistan or tuvalu or uganda or ukraine or uzbekistan or vanuatu or venezuela or vietnam or "west bank" or yemen or yugoslavia or zaire or zambia or zimbabwe).mp.	531582
94	(kabul or Porto-Novo or Hogbonou or Adjace or Cotonou or Kutonu or Ouagadougou or Ouaga or Bujumbura or Usumbura or Phnom Penh or Bangui or Bangi or N'Djamena or Ndjamena or Fort Lamy or Moroni or Kinshasa or Asmara or Asmera or Addis Ababa or Addis Abeba or Banjul or Bathurst or Conakry or Bissau or Port-au-Prince or Pyongyang or Monrovia or Antananarivo or Tananarive or Tana or Lilongwe or Bamako or Maputo or Lourenco Marques or Kathmandu or Niamey or Kigali or Freetown or Free-town or Mogadishu or Xamar or Hamar or Muqdisho or Maqadishu or Juba or Dodoma or Dar es Salaam or Lome or Kampala or Harare or Salisbury or Yerevan or Dhaka or Dacca or Thimphu or Thimbu or Sucre or Charcas or La Plata or Chuquisaca or La Paz or Praia or Yaounde or Jaunde or Brazzaville or Yamoussoukro or Cairo or Accra).mp.	24386
95	(Tegucigalpa or Tegus or New Delhi or Jakarta or Nairobi or South Tarawa or Tarawa Teinainano or Pristina or Prishtina or Bishkek or Pishpek or Frunze or Vientiane or Maseru or Nouakchott or Palikir or Chisinau or Kishinev or Rabat or Nay Pyi Taw or Naypyidaw or Nepranytau or Naypyitaw or Kyetpyay or Pyinmana or Kyatpyay or Pyinmana or Yangon or Rangoon or Managua or Abuja or Lagos or Islamabad or Port Moresby or Moresby or Pom Town or Manila or Apia or Dakar or Honiara or Jayawardenepura or Jayewardenepura or Khartoum or Mbabane or Embabane or Lobamba or Damascus or Dushanbe or Dyushambe or Stalinabad or Dili or Kyiv or Kiev or Tashkent or Toshkent or Port Vila or Hanoi or Ha Noi or Sana'a or Sanaa or Sana or Lusaka or Ulaanbaatar or Ulan-Bator or Luanda or Tbilisi or Amman).mp.	21747
96	(("under developed" or underdeveloped or low income or middle income or developing or less developed or "third world" or poor or LMI or LLMI or LAMI) adj1 (countr* or nation* or economy or economies)).mp.	128848
97	(("resource limited" or "low resource") adj1 (countr* or setting*)).mp.	7852
98	("less resourced communit*" or LMIC or LMICs).mp.	2416
99	or/23-98	1391627
100	3 and 22 and 99	2565
101	exp animals/ not humans/	4448581
102	100 not 101	2551
